# CTLA-4 in Regulatory T Cells for Cancer Immunotherapy

**DOI:** 10.3390/cancers13061440

**Published:** 2021-03-22

**Authors:** Navid Sobhani, Dana Rae Tardiel-Cyril, Aram Davtyan, Daniele Generali, Raheleh Roudi, Yong Li

**Affiliations:** 1 Department of Medicine, Section of Epidemiology and Population Sciences, Baylor College of Medicine, Houston, TX 77030, USA; dcyril@bcm.edu; 2 Atomwise, 717 Market St, San Francisco, CA 94103, USA; aram@atomwise.com; 3 Department of Medical, Surgery and Health Sciences, University of Trieste, 34147 Trieste, Italy; dgenerali@units.it; 4 Department of Medicine, University of Minnesota Medical School, Minneapolis, MN 55455, USA; Roudi002@umn.edu

**Keywords:** CTLA-4, T_reg_ cells, immune checkpoint inhibitors, CD28, antigen-presenting cells

## Abstract

**Simple Summary:**

In the fight against cancer, immunotherapies have given great hope after encouraging results in clinical investigations showing complete remission in some patients with melanoma. In fact, directing the immune system against cancer has been a very innovative strategy fostered during the past three decades. Despite this fact, the disease is serious, the mortality is still very high, and only a minority of patients are responsive to immunotherapies. Therefore, there is a need for a better understanding of the molecular mechanisms of resistance to immune checkpoint inhibitors such as antibodies against cytotoxic T-lymphocyte-associated protein 4 (CTLA-4). In this article, we discuss the molecular mechanism of CTLA-4 in T regulatory cell inhibition, while highlighting the knowledge gap.

**Abstract:**

Immune checkpoint inhibitors (ICIs) have obtained durable responses in many cancers, making it possible to foresee their potential in improving the health of cancer patients. However, immunotherapies are currently limited to a minority of patients and there is a need to develop a better understanding of the basic molecular mechanisms and functions of pivotal immune regulatory molecules. Immune checkpoint cytotoxic T-lymphocyte-associated protein 4 (CTLA-4) and regulatory T (T_reg_) cells play pivotal roles in hindering the anticancer immunity. T_reg_ cells suppress antigen-presenting cells (APCs) by depleting immune stimulating cytokines, producing immunosuppressive cytokines and constitutively expressing CTLA-4. CTLA-4 molecules bind to CD80 and CD86 with a higher affinity than CD28 and act as competitive inhibitors of CD28 in APCs. The purpose of this review is to summarize state-of-the-art understanding of the molecular mechanisms underlining CTLA-4 immune regulation and the correlation of the ICI response with CTLA-4 expression in T_reg_ cells from preclinical and clinical studies for possibly improving CTLA-4-based immunotherapies, while highlighting the knowledge gap.

## 1. Introduction

Globally, cancer remains the leading cause of mortality and morbidity, with nearly 9 million deaths every year [1]. Early diagnosis and advances in cancer treatment have improved the survival of cancer patients, but there were more than 1.7 million new cases of cancer in the United States in 2019 [1]. A considerable percentage of these patients manifested drug resistance, metastasis, and recurrence [2].

A promising paradigm in the dilemma and challenge of cancer therapy is immunotherapy, and the T cell population has generated considerable enthusiasm among scientists due to its ability to kill malignant tumor cells directly [3].

There are two major types of T cell: Conventional adaptive T cells (including helper CD4+ T cells [Th1, Th2, Th17, Th9, and Tfh], cytotoxic CD8+ T cells, memory T cells, and regulatory CD4+ T cells [T_reg_]) and innate-like T cells (including natural killer T cells, mucosal associated invariant T cells, and gamma delta T cells (γδ T cells)) [4]. The CD4+ T cells subset can target malignant tumor cells using different approaches, either by directly killing tumor cells or indirectly modulating tumor microenvironments (TME) [5,6]. These cells can increase the response of cytotoxic T cells (CTL) and quality of B cells [7]. The major killers of tumor cells are cytotoxic CD8+ T cells [8].

Innate-like T cells, representing one of the major groups of T cells, can be grouped into natural killer T cells (NKT cells), mucosal associated invariant cells (MAIT), and gamma delta T cells (γδ T cells) [9,10,11]. During development, innate-like T cells, called innate lymphoid cells (ILCs)-Natural Killer (NK) cells, acquire an effector function, whereas conventional T cells remain in a naive state [12]. The first group, NKT cells, express T-cell receptors (TCRs) and cell surface markers of NK cell lineages [13]. They are involved in the recognition of glycolipid antigens and present them to antigen-presenting cells (APCs) in the context of major histocompatibility complex (MHC) class I-associated protein CD1d [14]. T cells with γδ expression, representing the first layer of defense, constitute nearly 2% of the T cell population in peripheral blood and secondary lymphoid organs, while they are mainly found in the epithelia of the skin, gut, lung, and other organs [15,16]. Another group of innate-like T cells, called MAIT cells, constitute approximately 5% of all T cells and have considerable similarities to NKT cells [17,18].

T_reg_ cells are one of the most fascinating immunosuppressive subsets of CD4+ (CD25+) T cells, mainly represented by master transcription factor 3 (FOXP3), and they account for nearly 5% of the total CD4+ T cell population under normal conditions [19]. T_reg_ cells increase dramatically in response to the early stages of malignant tumor initiation and growth [20]. In the tumor microenvironment, T_reg_ cells can suppress the immune system activity of cytotoxic T lymphocytes (CTLs) [21]. A panel of immune-modulatory receptors expressed on the T_reg_ cell population includes cytotoxic T lymphocyte antigen 4 (CTLA-4), the vascular endothelial growth factor receptor (VEGFR), and programmed cell death protein 1 (PD1) [22]. CTLA-4 is expressed on activated T and T_reg_ cells [23,24] https://paperpile.com/c/d61gxv/defR (accessed on 5 February 2021). Atkins et al. showed that an immune checkpoint blockade of CTLA-4 improved the survival rate of renal cell carcinoma, melanoma, non-small cell lung cancer (NSCLC), and head and neck squamous cell cancer [25]. This protein was the second receptor of the T-cell costimulatory ligand CD80/86 and, therefore, an immune checkpoint whose function is critical for downmodulating the immune response. In contrast to the first receptor (CD28), which is antigen-dependent, CTLA-4 is antigen-independent [26]. In 2011, ipilimumab was the first immunotherapy drug targeting CTLA-4 to receive FDA approval to treat late-stage melanoma [27]. This approval came after encouraging results of a large randomized phase III clinical trial improving patients’ survival compared to standard therapy. Since then, several immunotherapies targeting the PD-1/PD-L1 axis have received FDA approval to treat multiple types of cancer [27].

This review will describe the mechanisms of CTLA-4 immune checkpoint inhibition, the role of T_reg_ cells in tumorigenesis, and how anti-CTLA-4 antibodies can provoke an alteration in the expression of CTLA-4 on T_reg_ cells while exerting anti-cancer therapeutic activity.

## 2. Mechanism of CTLA-4 Immune System Inhibition

A better understanding of the biological mechanisms and functions of negative and positive co-stimulatory molecules has been shown to be essential for improving current and potentially new CTLA-4 or Programmed Cell Death 1 (PD-1) inhibitors for anti-cancer immunotherapies.

Once bound to B7-1 (CD80) or B7-2 (CD86), CTLA-4 switches-off antigen-presenting cells [28]. CTLA-4 was immediately increased after T-cell receptor (TCR) engagement, reaching its highest level of expression as a homodimer at 2–3 days after the activation of conventional CD4+ and CD8+ T cells [29,30]. CTLA-4 competes with costimulatory molecule CD28 for the CD80/86 ligands CD80 and CD86, for which it has a higher affinity and avidity [31,32]. It is necessary to inhibit interactions with both CD80 and CD86 with antibodies to optimally block the CD28-dependent proliferation of T cells in an allogenic mixed lymphocyte reaction stimulated with B lymphoblastoid cell lines. Since both CD80 and CD86 exert a positive costimulatory signal through CD28, the role played by CTLA-4 in the competitive inhibition of CD28 is important for attenuating T-cell activation, thereby fine-tuning the immune response [33]. Rapid binding kinetics with a very fast dissociation rate constant (k_off_) of both CTLA-4 and CD28 to CD80 has been observed (k_off_ ≥ 1.6 and ≥0.43 s^−1^) [34], which permits their instant competition. The function of T cells can be suppressed by T_reg_ cells through multiple mechanisms [35]. T_reg_ cells constitutively express CTLA-4 on their suppressive functions. CTLA-4-expressing T cells (T_reg_ or activated conventional T cells) have been shown to lower levels of CD80/86 costimulatory molecules available on APCs by CTLA-4-dependent sequestration via trans-endocytosis [36]. This event can negatively regulate the proliferation of non-T_reg_ T cells, as well as the production of cytokines.

RAG2-deficient mice reconstituted with CTLA-4-deficient bone marrow developed lethal inflammation of multiple organs and died around 10 weeks after reconstitution, whereas control mice (reconstituted with normal bone marrow) were healthy. Intriguingly, the mouse chimeras reconstituted with a mixture of normal and CTLA-4-deficient bone marrow remained healthy, without developing any disease [37]. The authors concluded that the disease observed in CTLA-4^−/−^ mice is not due to a T cell autonomous defect and that CTLA-4 triggering on normal T cells produces factors inhibiting the disease induced by CTLA-4-deficient T cells. It has been shown that mice selectively deficient in CTLA-4 in T_reg_ cells (Foxp3+) develop systemic lymphoproliferation and fatal T cell-mediated autoimmune disease, indicating that T_reg_ cells critically require CTLA-4 to suppress immune responses and maintain immunological self-tolerance [38,39].

Additionally, after T-cell activation by TCR, CTLA-4 within intracellular compartments is immediately transported to the immunologic synapse [40]. The stronger the TCR signaling, the more CTLA-4 transported to the immunological synapse [40]. After reaching the synapse, CTLA-4 becomes stable through its binding to the CD80 and CD86 ligands, leading to its accumulation and effective out-competition against CD28 [28]. Differences in both the affinity and avidity in ligand-binding cause selective CD28 or CTLA-4 recruitment to the immunological synapse. The major ligand leading to CTLA-4 localization in the synapse is CD80, while for CD28, it is CD86 [28]. In this way, CTLA-4 attenuates the positive co-stimulation of CD28, thereby limiting the downstream signaling of CD28, which is primarily achieved through PI3K and AKT [41,42]. This mechanism allows a fine-tuning of TCR signaling and therefore T-cell activity. The negative co-stimulation of CTLA-4 is intrinsically linked to CD80/86 and CD28 positive co-stimulations. CTLA-4 mainly regulates T cells at priming sites (e.g., gut or lymphoid organs such as spleen and lymph nodes). Since CTLA-4 plays a crucial function in the activation of T cells, its negative co-stimulation plays a critical role in tolerance. As a matter of fact, the biallelic genetic *Ctla-4* deletion in mice leads to their death at 3–4 weeks of age because of pronounced lymphoproliferation with multi-organ lymphocytic infiltration and tissue destruction, particularly with pancreatitis and myocarditis [43,44,45]. Mice lethality can therefore be prevented by normal T cell factors. Several groups foster the idea that extrinsic cell suppressive functions of CTLA-4 are mainly mediated through T_reg_ cells [38,46]. Others support the idea that CTLA-4’s ability to inhibit T cells is T_reg_ cell-independent [47,48]. An argument for the first line of thought is that a particular loss of CTLA-4 in T_reg_ cells was enough to induce abnormal T-cell activation and autoimmunity [38,49]. In fact, Wing et al. showed that the loss of CTLA-4 in T_reg_ cells was capable of hyper producing immunoglobulin E, systemic lymphoproliferation, fatal T cell-mediated autoimmune disease, and powerful tumor immunity [38]. After losing the CTLA-4-expressing subpopulation, the T_reg_ cells were not capable of exerting their T cell suppressive functions; in particular, they were not able to down-modulate the dendritic cell expressions of CD80 and CD86 [38]. It must be noted that the lack of CTLA-4 in T_reg_ cells also leads to an aberrant expression and expansion of T_conv_ cells, which can cause the latter cells to infiltrate and fatally damage nonlymphoid tissues and cells [49]. Therefore, CTLA-4 in T_reg_ cells is also needed to prevent the accumulation of T cells that may harm vital organs.

As a hypothetical molecular biology explanation, it is possible that T_reg_ cells with CTLA-4 may limit the availability of CD80/86 ligands for the positive co-stimulation of CD28 in effector T cells. Through such a mechanism, the CTLA-4 would indirectly and cell-extrinsically dampen T-cell activation. It is also known that CTLA-4 on effector T cells can trans-compete for CD80/86 ligands [50]. Another mechanism by which CTLA-4 can lower the total availability of CD80/86 ligands is through APC-mediated trans-endocytosis of CD80/86 ligands [36]. The last two mechanisms explain how CTLA-4 could prevent anti-cancer immune reactions without the need for T_reg_ cells. Overall, it is noteworthy that these mechanisms are not yet fully understood and each contribution remains elusive in the context of cancer immunity and drug design.

Furthermore, unexpectedly, the depletion of CTLA-4 from a T_reg_ cell population of adult mice conferred resistance to autoimmune encephalomyelitis (EAE) and did not enhance anti-tumor immunity [51]. This was accompanied by an expansion of functional CTLA-4-deficient T_reg_ cells expressing immunosuppressive molecules (IL-10, LAG-3, and PD-1) capable of protecting them from EAE, demonstrating that CTLA-4, in addition to previously described mechanisms of action, has a T_reg_-intrinsic effect in limiting T_reg_ expansion.

Additionally, since CTLA-4 expression has been correlated with the TCR signal strength, high T_reg_ cell and CTLA-4 expressions are concomitant [52,53]. The inhibition efficacy of any cell by CTLA-4 depends on the affinity between the major histocompatibility complex (pMHC) ligand and its TCR. The higher the affinity of TCRs, the more those cells can be inhibited through CTLA-4 [54,55]. Additionally, the induction of CTLA-4 also restricts CD4+ T-helper clonal expansion. Ultimately, through such a mechanism of action of CTLA-4, the TCR signal is fine-tuned in response to specific immunological threats.

Furthermore, a number of structures of the extracellular domain of human CTLA-4 are available in Protein Data Bank (PDB), including apo structures and various complexes. The very first structure of CTLA-4 was determined using solution NMR spectroscopy (PDB ID: 1AH1), revealing an Ig-like V (variable)-type domain, where two beta-sheets of the V-fold are connected by two disulfide bonds (21 to 94 and 48 to 68) [56]. Another apo structure of CTLA-4 was later published in the physiological dimeric state (PDB ID: 3OSK) [57]. CTLA-4 binds its native ligands CD80 and CD86 at the A ‘GFCC’ face, which contains a number of charged residues that are highly conserved between CTLA-4 and CD28 (and across species). A key role in these interactions is also played by the _99_MYPPPY_104_ loop connecting F and G strands [56]. The structures of CTLA-4 with CD80 and CD86 (PDB IDs: 1I8L and 1I85) manifested a mostly convex binding surface at CTLA-4, free of any notable cavities that could have been targeted with traditional small-molecule campaigns [58,59]. It is also interesting to note that while the CD80-bound conformation of CTLA-4 is very similar to the apo form, CD86 binding requires some structural rearrangement, most significantly, in the FG loop [57,58,59]. Finally, several structures of CTLA-4 bound to monoclonal antibodies have also recently been reported (PDB IDs: 5GGV, 5TRU, 5XJ3, and 6RP8) [60,61,62]. These structures reveal that ipilimumab and tremelimumab directly compete with CD80 and CD86 at their binding surface, sterically displacing and preventing their interactions with CTLA-4. Moreover, subtle differences in the CTLA-4 structure, such as a slightly larger distance between G and F stands, and extended interactions of antibodies with non-conserved residues on the opposite side of the FG loop, enable selectivity between CTLA-4 and CD28 [61]. Interestingly, the amino acid sequence of the intracellular tail of CTLA-4 is conserved in 100% of all mammalian species, meaning that its intracellular domain must have an important role in the inhibition of T-cell activation [63,64]. In fact, the inhibitory functions of CTLA-4, by competing with CD28 for CD80 and CD86 or through its transmission of negative signals, can be accomplished because of its intracellular domain, but such a downstream mechanism of CTLA-4 signal transduction deserves further investigations [64,65]. Based on the primary amino acid sequence of the CTLA-4 cytoplasmic region, there are two potential binding sites for Src homology domain 2 (SH2) and an SH3 potential binding motif [66]. CTLA-4 was found to be capable of becoming associated with SH2-containing tyrosine phosphatase-2 (SHP-2) through the SH2 domain of SHP-2. Such an association resulted in phosphatase activity against Ras regulatory protein p52SHC [67]. Therefore, CTLA-4 might be able to start a signal transduction cascade leading to the dephosphorylation of TCR-associated kinases or substrates.

While the antitumor activity and clinical benefits of antibodies such as ipilimumab that block CTLA-4 interactions with ligands have been demonstrated [61], it is always desirable to have bioavailable and cheaper options in the form of small molecules or peptides. In cases of traditionally undruggable targets, such as CTLA-4, where no suitable small-molecule binding pockets can be immediately identified at the ligand-binding interface, peptide drugs can present a viable alternative. Like antibodies, peptides can achieve a high affinity and specificity by capturing a larger interaction area with the target. At the same time, they are easier to synthesize and have greater tissue penetration due to their smaller size compared to the antibodies. Moreover, peptides have recommended themselves in a variety of therapeutic areas, including cancer [68,69]. In addition, targets similar to CTLA-4 can be amenable to less-standard small molecule campaigns. One such approach is allosteric modulation. In this case, a small molecule bound to a distant site can activate or inhibit the protein function or its interactions with other molecules as a result of structural changes that it induces at a distance [70]. However, for CTLA-4, such sites still have to be determined through either experimental or computational techniques [71,72].

## 3. Regulatory T Cells and Anticancer Immunity

### 3.1. First Insights into T_reg_ Cells

T_reg_ cells are a population of CD4 T cells constitutively expressing CTLA-4. They are crucial for both immune-oncology and autoimmunity, as we will describe in this review. The focus of this article is on the CTLA-4-positive population of T_reg_ cells in cancer. After T_reg_ cells were discovered for the first time in the CD4+ CD25+ T cell subpopulation in 1995 [73], mutations of *FOXP3* recapitulated the impaired formation or improper function of T_reg_ cells, causing an immune dysregulation syndrome in mice, termed polyendocrinopathy enteropathy X-linked syndrome, which ultimately leads to multiple autoimmune disorders [74]. Corroborating the importance of T_reg_ cells for a functional immune response, mice carrying spontaneous alterations of *Foxp3*—that ultimately lacked T_reg_ cells—died due to systemic autoimmunity [75,76]. As expected, the external expression of FOXP3 bestowed naïve CD4+ T cells (T_conv_, without T_reg_ cells) with the same immune-suppressive capacity typical of T_reg_ cells. Therefore, FOXP3 is a master transcription factor that regulates T_reg_ cell phenotypes and their function as immunosuppressants. The role of T_reg_ cells in cancer is mainly observed at inflammatory sites, where they migrate and inactivate different types of effector T cells, such as CD4^+^ T helper (T_H_) cells and CD8^+^ cytotoxic T cells (CTLs) [77,78,79,80]. As a consequence, intervening in this activity of T_reg_ cells could induce the immune system in the fight against cancer.

### 3.2. Inhibitory Effects of Treg Cells on APC

T_reg_ cells represent a crucial component of the immune system, being essential for controlling self-tolerance, and thereby play essential roles in various medical conditions. T_reg_ cells have a crucial role in the suppression of the immune response in cancer [73,75,81,82,83,84,85]. T_reg_ cells inhibit APC by three main mechanisms: (1) Depleting immune-stimulating cytokines [86,87,88,89]; (2) producing immunosuppressive cytokines (like TGF-β, IL-10, and IL-35); and (3) constitutively expressing CTLA-4. T_reg_ cells express Interleukin 2 (IL2) receptors that bind to IL2, thereby limiting the amount of this cytokine available for T_conv_ cells [90,91]. As a consequence, the constitutive expression of CTLA-4 blocks the priming and activation of T_conv_ cells to APCs [38,92].

**Figure 1:**
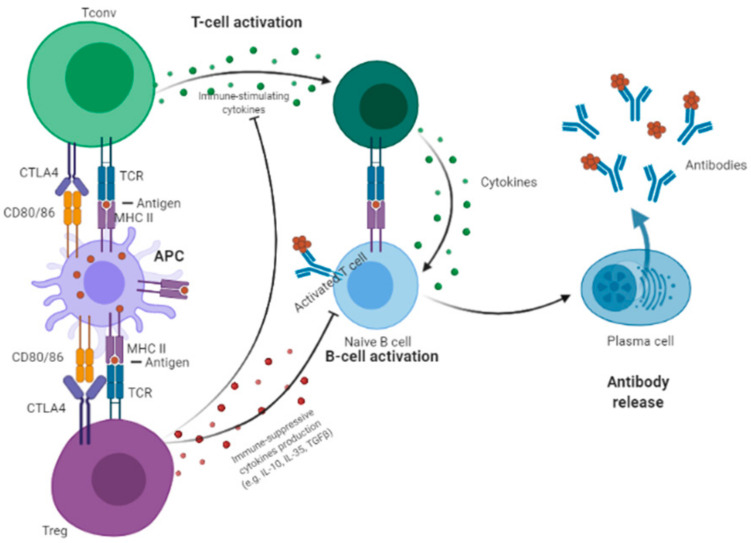
Regulatory T (T_reg_) cells inhibit antigen-presenting cells (APC) by three main mechanisms: (1) Depleting immune-stimulating cytokines; (2) producing immunosuppressive cytokines (e.g., TGF-β, IL-10, and IL-35); and (3) constitutively expressing CTLA-4, which blocks the priming and activation of naïve CD4+ T (T_conv_) cells to APCs.

Figure 1 summarizes the role of CTLA-4 in T_reg_ cells modulating T_conv_ activation.

T_reg_ cells block normal protective immune-surveillance and inhibit the antitumor immune response in cancer patients. Thereby, if T_conv_ cells are like tumor suppressors, T_reg_ cells could be considered as oncogenes because they are suppressing antitumor immunity [81,82,93,94], although the definitions of oncogenes and tumor suppressors refer to genes in tumors that, when expressed, cause or prevent cancer, respectively [95]. Likewise, CTLA-4 and PD-1 immune checkpoints, since they block the immune system’s recognition of cancer cells, could also be comparable as tumor suppressors.

### 3.3. Conflicting Roles of T_reg_ Cells in Malignant Tumors

The role of T_reg_ cells in immunoncology was discovered by two Japanese groups in 1999 [93,94]. The two groups independently reported that anti-CD25 antibodies, capable of depleting CD4+CD25+ T_reg_ cells, led to higher tumor rejection and retarded tumor growth in normal and T cell reconstituted nude mice [93,94]. CD25 is the α chain of the interleukin-2 receptor. Onizuka et al. showed that a single dose (less than 0.125 mg) of anti-CD25 was capable of causing the regression of multiple tumors derived from four different inbred mouse strains (five leukemias, myeloma, and two sarcomas) [93]. Similarly, Shimizu et al. showed that the elimination of CD25-expressing T cells caused a powerful immune response in syngeneic tumors in mice, leading to tumor regression within 1 month, thereby allowing the host to survive > 80 days [94]. Among CD4+ T cells, the percentage of T_reg_ cells is higher in the blood of cancer patients compared to that of healthy individuals [83,96,97]. Expectedly, the relatively higher T_reg_ cell levels in the tumor microenvironment correlated with a poor prognosis in various cancer types, such as melanoma and non-small cell lung, ovarian, and gastric cancers [82,83]. The T_reg_ cell population is not large in the periphery blood of cancer patients compared with the TME, implying that T cells’ interaction with tumor cells is important [97]. On the contrary, in certain tumors, such as colorectal cancer (CRC), a high level of FOXP3+ T cells is correlated with a better prognosis [98]. This is because the accumulation of FOXP3+ occurs together with inflammatory cytokines, possibly implying that T_reg_ cells play a role in repressing tumor inflammation. It was brought to light that two populations of FOXP3 (+) CD4 (+) T cells had distinct roles in controlling the prognosis of CRCs, contributing in opposing ways. FOXP3 (hi) T_reg_ cells are correlated with worse survival, whereas FOXP3 (lo) non-T_reg_ T cells are correlated with better survival. This is possibly because the FOXP3+ (lo) non-T_reg_ T cell population leads to an inflammatory TME against the tumor. In fact, it was observed that FOXP3+ non-T_reg_ T cells in CRCs are correlated with high levels of tumor necrosis factor (TNF), IL2, and TGFβ [96]. Depleting FOXP3 (hi) T_reg_ cells from tumor tissues could be deployed to increase the antitumor immunity to treat CRC or other cancers, whereas other strategies enhancing the levels of FOXP3(lo) non-T_reg_ T cells could also be used to suppress or prevent tumorigenesis [96].

There are conflicting reports regarding the prognostic value of tumor-infiltrating T_reg_ cells. Shang et al. demonstrated that FOXP3+ T_reg_ cells are correlated with shorter overall survival in breast, hepatocellular, gastric, melanoma, renal, and cervical cancers, and longer overall survival in head and neck, colorectal, and esophageal cancers, whilst they display no correlation for pancreatic and ovarian cancers [99].

In conclusion, T_reg_ cells inhibit anti-cancer immunity, blocking the immune surveillance of tumors, which ultimately leads to cancer spreading [81,82,83,93,94]. Immunosuppressive T_reg_ cells, producing cytokines, are observed in both human chronic inflammatory disease and cancers, where they promote tumorigenesis through a mechanism similar to that of chronic inflammation [48,100,101]. The depletion of T_reg_ cells in mice is capable of promoting lymphocyte recruitment and as a consequence, a decrease in the tumor growth rate and the presence of high endothelial venules, indicating destruction of the tumor tissues [102,103].

### 3.4. T_reg_ Cells and the Tumor Microenvironment

The TME is mainly comprised of a subpopulation of T_reg_ cells called bona fide T_reg_ cells that enhance the expression of immunosuppressant molecules such as CTLA-4 and T-cell immunoreceptors with Ig and ITIM domains (also called TIGIT), whose expression is very low in naïve T_reg_ cells [83,96,104]. A transcriptome analysis of 15 human lung cancer samples and 14 colorectal cancer samples demonstrated that tumor-infiltrating T_reg_ cells have very high levels of different T_reg_ activation markers, such as T cell immunoglobulin mucin receptor 3 (HAVCR2), glucocorticoid-induced TNFR-related protein (GIRT), lymphocyte-activation gene 3 protein (LAG3), and inducible T cell co-stimulator (ICOS). Interestingly, this phenotype was not observed in peripheral blood samples from the same patients, whose expression levels in the blood remained the same. This could indicate that T_reg_ cells become activated in TME, where they exert their immune-suppressive functions [105].

### 3.5. Cross-Talk between T_reg_ Cells and the Tumor Microenvironment

It has recently been shown that adenosine produced by apoptotic T_reg_ cells present within the TME exerts higher immunosuppressive effects compared to live T_reg_ cells [21,106]. A weak NRF2-associated antioxidant pathway leads to a vulnerable system against reactive oxygen species in TME, possibly causing apoptosis in T_reg_ cells, which is a process that has been shown to convert high ATP levels into adenosine through T_reg_ cell-expressed ectoenzymes CD39 and CD73. In turn, the resulting abundance in adenosine engages purinergic adenosine A2A receptors (also known as ARORA2A), which is a family of G protein-coupled receptors with seven transmembrane alpha helices whose function is to regulate the oxygen demand and increase vasodilatation, as well as suppress immune cells. Apoptotic T_reg_ cells use the A2A pathway to suppress immune cells [21,106]. The mechanism postulated to explain the activation of T_reg_ cells in TME is that proliferating and dying tumor cells have loads of self-antigens, which are best recognized through T_reg_ cells and thereby become activated in TME [107]. Another explanation comes from results from mice experiments of two research groups showing that immune dendritic cells expressed in mice tumors activate T_reg_ cells in a TGFβ-dependent manner [107,108]. T_reg_ cells recognize specific self-antigens and can become clonally expanded in TME [109,110]. T_reg_ cells typically have higher affinity TCRs for self-antigens than T_conv_ cells and therefore, should be predominantly activated, even when in competition with T_conv_ cells. It must be stated, however, that these data come from animal studies and T_reg_ cells induced by TFGβ have not yet been fully demonstrated in humans. As for the epigenetic profile of tumor-infiltrating T_reg_ cells, very little is understood [111,112,113]. Epigenetic studies of T_reg_ cells are limited and future studies could shed more light on the subject, in order to better understand the origin and mechanisms of activation of T_reg_ cells. T_reg_ cells move to the TME by chemotaxis via chemokines and their receptors, such as CXCL12-CXCR4, CCL5-CCR5, CCL22-CCR4, and CCL1-CCR8 [83,105,114,115,116,117,118]. Blocking such chemotactic signals can reduce the accumulation of T_reg_ cells inside tumors [119]. Such chemokines are produced in the TME by the tumor and/or macrophages [83,105,114,115,116]. Additionally, some chemokines, such as CCL1 and CCL22, can be produced within tumors by exhausted or dysfunctional CD8+ T cells [119,120]. Therapies targeting chemokines could be considered to lower the T_reg_:T_conv_ ratio in the tumor microenvironment, in order to produce more T_conv_ and less T_reg_ cells. Cancers engage various immune escape mechanisms that can be dependent on specific tumor intrinsic factors. In fact, alterations in tumor suppressor PTEN; Liver Kinase B1 (LKB1); or oncogenes WNT/β-catenin, KRAS, or basic leucine zipper transcriptional factor ATF-like 3 (BATF3), affect effector T-cell recruitment to the tumors [121,122,123,124,125]. On the contrary, tumor hyper-activation of FAK leads to a recruitment of T_reg_ cells, together with chemokine-driven CD8+ T cell exhaustion or poor infiltration within the tumor [126,127]. In fact, Jiang et al., using tissues from pancreatic ductal adenocarcinoma (PDAC) patients, observed that FAK was elevated and correlated with high levels of fibrosis and poor CD8+ cytotoxic T cell infiltration, which are signs of an immune-suppressive TME. The use of a FAK inhibitor (VS-4718) substantially limited tumor progression and doubled the survival of a humanized mice model of PDAC [126]. In squamous cell carcinoma (SCC) cells, FAK was shown to drive the exhaustion of CD8+ T cells and recruitment of T_reg_ cells in TME through the regulation of chemokines/cytokines and ligand-receptor networks (such as Ccl5/Ccr5), ultimately permitting tumor growth. FAK kinase inhibitor VS-4718 drove T_reg_ cell depletion and promoted the anti-tumor response of CD8+ T cells [127].

### 3.6. Treg Cells and Nonself Antigens

At the location of tumor cells, there are two types of antigens recognized by T_reg_ cells: Shared antigens and neoantigens. The first ones arise from highly or aberrantly expressed endogenous proteins encoded by the germ line. The second ones derive from either abnormal self-proteins formed from somatic genetic alterations or from oncogenic viral proteins. Experiments with animals have shown that T_reg_ cells primed to nonself antigens increased the affinity of CD8+ T cells, most likely by the inhibition of T cells carrying TCRs with low-avidity to antigens [128]. APCs can render CD8+ T cells targeting self-antigens self-tolerant through the control of T_reg_ cells [129]. In fact, using human T cells in vitro, the authors showed that T_reg_ cells were able to make the self-reactive human CD8+ T cells anergic upon antigen stimulation. In addition, they observed the proliferative activity of self-antigen-specific T cells in CTLA-4+ and CTLA-4- fractions. The CTLA-4+ fraction was highly proliferative, had a low expression level of BCL2, and was prone to death upon self-antigen stimulation. On the contrary, T_reg_ cells did not suppress non-self-specific CD8+ T cells [129]. Therefore, T_reg_ cell-mediated immunosuppression could be more effective in shared antigen-expressing tumors compared to those expressing neoantigens. This could be a reason why tumors expressing neoantigens respond better to immune checkpoint blocking than tumors with a low mutational burden [130,131]. One of the major aims of immunotherapy research is to understand why some cancer patients respond very well to immune checkpoint inhibitions while others do not, as well as discovering new biomarkers useful for just-in-time determination of treatment-responsive patients, before administrating immunotherapies.

## 4. Correlation between Anti-CTLA-4 Treatment and Its Effect on T_reg_ Cells

The anti-CTLA-4 monoclonal antibody ipilimumab (Yervoy, Bristol-Meyers Squibb) gained FDA approval in March 2011 for the treatment of advanced melanoma, which is the most dangerous type of skin cancer, after a large randomized phase III clinical trial consisting of 676 patients demonstrated that ipilimumab improved the overall survival (OS) of melanoma patients who did not respond to standard therapy. In fact, the median OS in 403 patients randomly assigned to receive 3 mg/kg ipilimumab with an investigational vaccine made of HLA-A*01201-restricted glycoprotein 100 with incomplete Freund’ adjuvant was 10.0 months (gp100, 95% Confidence Interval [CI], 8.5–11.5) vs. 6.4 months observed for 136 patients treated with gp100 only (Hazard Ration [HR] for death = 0.68; *p* = 0.001). In total, 137 patients were treated with ipilimumab alone and had an OS of 10.1 months vs. 6.4 months for the gp100 alone group (95% CI, 9.0–13.8; HR for death = 0.66, *p* = 0.003) [132]. After its approval, the drug was added as a category 1 recommendation in the National Comprehensive Cancer Network (NCCN) guidelines for the systemic treatment of advanced or metastatic melanoma.

This clinical evidence shows that the antibody enhanced the ability of the immune system to attack cancer through CTLA-4 inhibition. It must be mentioned that adverse events occurred in 10–15% of patients treated with ipilimumab alone compared to patients treated with gp100 only [132].

In 2014, another pivotal phase III clinical trial (CA184-024) including 502 metastatic melanoma patients tested ipilimumab. The current standard of care treatment for the disease is chemotherapy (decarbazine), which has not been shown to increase OS. Interestingly, the treatment of patients with 850 mg/m^2^ decarbazine with 10 mg/kg ipilimumab improved OS compared to an arm with only chemotherapy with the placebo. The OS of patients treated with ipilimumab plus decarbazine vs. decarbazine plus placebo was 47.3% vs. 36.3% at the first year, 28.5% vs. 17.9% at the second year, and 20.8% vs. 12.2% at the third year (HR for death with ipilimumab/decarbazine, 0.72; *p* < 0.001). The risk of progressing through the disease decreased by 24% when using ipilimumab/decarbazine vs. decarbazine/placebo (HR for progression, 0.76; *p* = 0.006). The ratios of the disease to control were similar for the two groups (33.2% for ipilimumab/decarbazine and 30.2% for decarbazome/placebo; *p* = 0.41). This study was important because it showed how ipilimuamb could be used as the first line treatment for metastatic melanoma [133]. The study tested a higher concentration (10 mg/kg) of ipilimuab than the approved 3 mg/kg [134]. Consequently, more adverse events were observed using higher doses of anti-CTLA-4, possibly because of CTLA-4 molecular degradation. In fact, CTLA-4 is needed to prevent immune-related adverse reactions and its degradation can be deleterious.

Interestingly, a recent report demonstrates that the immune-related Adverse Events (irAEs) of ipilimumab and alike result from the lysosomal degradation of CTLA-4 in T_reg_ cells. The study used the CTLA-4 mutant (Y201V), which is incapable of being recycled because it lacks interaction with the lipopolysaccharide (LPS)-responsive and beige-like anchor protein (LRBA). This indicates that the specific region of CTLA-4 is an essential mediator of CTLA-4 recycling. The investigators made antibodies targeting CTLA-4 (HL12 and HL32) that were not able to degrade the CTLA-4 of T_reg_ cells. In fact, in contrast to ipilimumab or TremeIgG1, the use of novel anti-CTLA-4 antibodies had no effect on the CTLA-4 level of T_reg_ cells in the same model. Additionally, HL12 and HL32 could more effectively lead to tumor rejection, with fewer irAEs in mice [135]. Such knowledge is useful for the generation of novel antibodies or molecules that could inhibit CLTA-4 without eliciting its degradation and could therefore be used in combination with other PD-1 or PD-L1 inhibitors with less toxicity.

Various studies show that consolidated or novel types of CTLA-4 therapies correlate with different expression levels of T_reg_ cells. Ji et al. showed that the treatment of mice with 0.25 mg anti-CTLA-4 monoclonal antibody correlated with a lower level of the CD25+Foxp3+ T_reg_ cell population (*p* < 0.05) [136]. Qu et al. observed that anti-CTLA-4 monoclonal antibodies enhanced IL36-stimulated antitumor activity by depleting T_regs_ in the tumor [137]. Mihic-Probst et al. showed that anti-CTLA-4 antibody ipilimumab, anti PD-1 antibody nivolumab, or pembrolizumab decreased the number of CD25+ T_reg_ cells [138]. Sun et al. observed that the number of T_reg_ cells decreased after treating mice with anti-CTLA-4 or anti PD-1 antibodies in an HPV16 E6/E7^+^ syngeneic mouse tumor model [139]. Kvarnhammar et al. showed that new IgG1 bispecific anti-CTLA-4 and anti-OX40 induced the activation of T cells and T_reg_ cell depletion in vitro and in vivo in the tumor [140]. Sharma et al., using samples from 19 melanoma, 17 prostate, and 9 bladder cancer patients treated with ipilimumab and 18 samples from melanoma cancer patients treated with tremelimumab, observed that the monoclonal antibodies depleted intratumoral FOXP3 T_reg_ cells in tumors [48]. Pai et al. devised a dual variable domain immunoglobulin of the anti-CTLA-4 antibody (anti-CTLA-4 DVD) possessing an outer tumor-specific antigen-binding site engineered to shield the inner anti-CTLA-4-binding domain. The latter only became available upon reaching the tumor after cleavage of the construct by proteases present in the tumor. In a preclinical tumor model, treatment with the anti-CTLA-4 DVD led to the depletion of tumor-resident T_reg_ cells, while preserving tissue-resident T_reg_ cells, resulting in an efficient antitumor response with a reduced multi-organ immune toxicity [141]. Morris et al. observed that anti-CTLA-4 antibodies IgG2a and IgG2b isotypes of 9D9 clone decreased the number of T_reg_ cells in syngeneic murine tumors of B78 melanoma and/or Panc02 pancreatic cancer [142]. Duperret et al. observed that, upon treatment with anti CTLA-4 in combination with a TERT DNA vaccine administered once a week for four rounds of immunization in C57BL/6 mice, the level of T_reg_ cells decreased within the tumors, while it remained unchanged within the peripheral blood [143]. Tang et al. observed, through IHC and quantitative real-time PCR, that the anti-CTLA-4 monoclonal antibody decreased the presence of T_reg_ cells in the mice tumor microenvironment, but not in peripheral lymphoid organs [144]. Son et al. showed that the anti-CTLA-4 antibody and radiotherapy suppressed CD25 T_reg_ cells in C57BL mice injected with lung cancer [145]. Schwarz et al. investigated the effect of using different doses of anti-CTLA-4 in the presence of T_reg_ cells in mice. They used a low dose of 0.25 mg CTLA-Ig antibody (LD, 10 mg/kg body weight), high dose of 1.25 mg CTLA-Ig antibody (HD, 50 mg/kg body weight), and very high dose of 6.25 mg CTLA-Ig antibody (VHD, 250 mg/kg body weight). T_reg_ cell levels decreased, independently of the doses [146]. Marabelle et al., using a combination of anti-CTLA-4 and anti-OX40 with CpG therapy, observed a reduction of T_reg_ cells in tumors [147].

Interestingly, Du et al. observed that anti-CTLA-4 antibodies are capable of efficiently inducing T_reg_ cell depletion and tumor regression in mice [148].

In contrast, several other groups reported an increase of T_reg_ cells in cancers after anti-CTLA-4 treatment. In fact, Sandi et al. observed that high dose treatment of anti-CTLA-4 increased the accumulation of T_reg_ cells in secondary lymphoid organs [149]. Kavanagh et al. observed that the anti-CTLA-4 antibody ipilimumab in four cohorts of patients increased T_reg_ cell levels in a dose-dependent manner. The drug was administered every 28 days [150]. Quezada et al. observed that a CTLA-4 blockade with GM-CSF combination immunotherapy in an in vivo B16/BL6 mouse model of melanoma led to a self-expansion of T_reg_ cells in tumors [47]. The reason for such discrepancies between the last four studies and the majority of studies described in the previous paragraphs remains unknown. A possible explanation could be that different subpopulations of T_reg_ cells were detected by the groups, such as bona fide and naïve Treg cells, or that the organisms’ TMEs of either animals or humans were different across the different experimental settings.

Of note, CTLA-4 has two opposing and crucial properties in cancer and autoimmunity. For self-tolerance it is important to have functional CTLA-4. Current antibodies developed against CTLA-4 have the property of reducing the levels of CTLA-4 by 50% by lysosomal degradation, which is directly responsible for their toxicity [135]. Therefore, since CTLA-4 is crucial for preventing autoimmunity, which is the major cause of irAE triggered by monoclonal antibodies such as ipilimumab and tremelimumab [135], new drugs should be developed considering such a gap. Encouraging results have already been produced by Zhang et al. HL12 and HL32 anti-CTLA-4 antibodies did not change the CTLA-4 level total or that in the T_reg_ cell fraction, while exerting powerful anti-CTLA-4-induced tumor inhibition [135]. Table 1 summarizes all the studies investigating anti-CTLA-4 therapies’ effect on T_reg_ cell levels.

Moreover, in clinical routines, it should also be considered that T cells are made of multiple subpopulations with their own peculiar effects. The modulation of T_reg_ cells and/or T_eff_ cells and pro-inflammatory responses is critical for cancer. An immunosuppressive state (increased T_reg_ and/or decreased T_eff_) may facilitate the growth and spread of abnormal cancer cells. Therefore, the T_reg_:T_eff_ ratio could be used in a clinical setting. The new checkpoint inhibitors attempt to pharmacologically modulate the T_reg_:T_eff_ ratio in the treatment of cancer therapy. However, in cancer progression, the expression of co-inhibitory molecules by tumors favors an imbalance in the tumor microenvironment toward an immune suppression status by increasing T_reg_ infiltration and decreasing T_eff_ activity [151]. On the contrary, the ratio of T_reg_:T_eff_ should be in favor of T_reg_ depletion and an increase of activated effector T cells, in order to potentiate an anti-tumor response [152]. Tremelimumab was shown to improve the proliferative response of T_eff_ and to abrogate the T_reg_ suppressive ability, suggesting that monitoring these populations may allow for the proper selection of responsive patients from those who would not obtain a benefit from immunotherapy [153]. With regards to the patients’ management, it seems to be crucial to understand and monitor the “ping-pong” effect produced by treatment of the T_reg_:T_eff_ ratio in the regulation of autoimmunity and anti-tumor immunity. Clinicians should pay attention to monitoring this effect in order to maintain an effective anti-tumor response and immune homeostasis preventing the onset of IRAEs [154].

## 5. Conclusive Remarks and Future Directions

In conclusion, most studies have shown that anti-CTLA-4 antibodies mainly depleted T_reg_ cells in cancers, whereas very few have observed that the number of T_reg_ cells increased or remained the same because of different experimental settings or in some cases, the design of their therapeutic agents. It is generally known that T_reg_ cells inhibit anti-cancer immunity, blocking the immune surveillance of tumors, ultimately leading to cancer growth. In our opinion, antibodies or small molecules that inhibit CTLA-4, but do not alter CTLA-4 levels in T_reg_ cells, could be innovative and ultimately more effective in eradicating cancer cells. In fact, such drugs would not cause the degradation of CTLA-4 and consequently, do not interfere with T_reg_ cells’ function in preventing autoimmunity. Consequently, the inhibition of CTLA-4 could be achieved without the degradation of CTLA-4 and adverse related events caused by toxicity. Testing their efficiency, together with other checkpoint inhibitors, such as anti-PD1 and anti-PD-L1, could further improve the therapy efficacy.

## Figures and Tables

**Table 1:** Effects of anti-CTLA-4 therapy on T_reg_ cells.

Reference	Anti-CTLA-4 Therapy and Samples	Effect on the Presence of T_reg_ Cells
Ji et al. 2020 [136]	In vivo investigated effect of administration of 0.25 mg anti-CTLA-4 monoclonal antibody on the CD25+Foxp3+ population in spleens and tumor tissues.	Decreased T_reg_ cells (*p* < 0.05) in tumor. It did not in spleen.
Qu et al. 2020 [137]	CTLA-4 monoclonal antibodies.	Decreased T_reg_ cells in tumors.
Probst et al. 2020 [138]	All patients received anti-CTLA-4 therapy and four received additional anti-PD1 therapy.	Decreased T_reg_ cells in tumors.
Zhang et al. 2019 [135]	In vivo anti-CTLA-4 therapy ipililumab and TremeIgG1 standard and HL12 and HL32 experimental antibodies.	Ipilimumab and TremeIgG1 downregulated cell-surface and total CTLA-4 levels in T_reg_ cells from spleen and lung. In contrast, HL12 and HL32 had no effect on CTLA-4 level of T_reg_ cells in the same model.
Sun et al. 2019 [139]	In vivo anti–CTLA-4 antibody.	Downregulation of T_reg_ cells in tumors of mice.
Kvarnhammar et al. 2019 [140]	CTLA-4 x OX40 bispecific antibody. ATOR-1015 was used in vivo.	Reduced the frequency of T_reg_ cells in vitro and at the tumor site in vivo.
Sharma et al. 2019 [48]	Nineteen melanoma patient, 17 prostate cancer patient, and 9 bladder cancer patient samples were treated with ipilimumab. Eighteen melanoma tumors were treated with tremelimumab.	mAbs depleted intratumoral FOXP3+ T_reg_ cells in tumors via Fc-dependent mechanisms.
Pai et al. 2019 [141]	Anti CTLA-4 DVD Ig tetravalent bispecific antibody-like antibody containing an Fc region and two pairs of variable domains joined in tandem by a short flexible linker.	Decreased T_reg_ cells in mouse tumors, but not in tissues.
Tang et al. 2019 [144]	Anti-CTLA-4 monoclonal antibody.	Increase of T_reg_ cells in tumors.
Morris et al. 2018 [142]	Anti-CTLA-4 (IgG2a and IgG2b isotypes of the 9D9 clone)	Decreased T_reg_ cells in tumors.
Duperret et al. 2018 [143]	Anti-CTLA-4 with a TERT DNA vaccine in vivo in C57BL/6 mice. Mice were immunized at 1-week intervals for a total of four immunizations.	Decreased T_reg_ cell frequency within the tumor. No decrease in peripheral blood.
Du et al. 2018 [148]	In vivo anti-CTLA-4 antibodies binding to human-like ipilimumab.	T_reg_ cell depletion.
Son et al. 2017 [145]	Anti-CTLA-4 antibody therapy and radiotherapy in vivo.	Suppression of T_reg_ cells in tumors.
Schwarz et al. 2016 [146]	In vivo anti-CTLA-4 low dose (0.25 mg), high dose (1.25 mg), and very high dose (6.25 mg) were given to mice.	CD25 T_reg_ cells were reduced independently of the doses.
Sandin et al. 2014 [149]	In vivo comparison of low-dose peritumoral and high-dose systemic CTLA-4 blockade therapy.	As opposed to low-dose therapy, high-dose systemic therapy stimulated accumulation of T_reg_ cells in secondary lymphoid organs. This could counteract immunotherapeutic benefit of CTLA-4 blockade.
Marabelle et al. 2013 [147]	In vivo anti-CTLA-4 and anti-OX40 with CpG.	Depleted T_reg_ cells in tumors.
Sandin et al. 2010 [149]	In vivo anti-CTLA-4 or anti-PD-1 with CpG therapy.	The combinations reduced numbers of T_reg_ cells at tumor site.
Kavanagh et al. 2007 [150]	In vivo anti-CTLA-4 antibody dose escalation.	Increased T_reg_ cells in tumors in a dose-dependent manner.
Quezada et al. 2006 [47]	In vivo CTLA-4 blockade and GM-CSF combination immunotherapy mice model B16/BL6 melanoma.	Led to self-expansion of T_reg_ cells in tumors.

## References

[1] Miller K.D.D., Fidler-Benaoudia M., Keegan T.H.H., Hipp H.S.S., Jemal A., Siegel R.L.L. (2020). Cancer statistics for adolescents and young adults, 2020. CA Cancer J. Clin..

[2] Siegel R.L., Miller K.D., Jemal A. (2019). Cancer statistics, 2019. CA Cancer J. Clin..

[3] Speiser D.E.E., Ho P.C.C., Verdeil G. (2016). Regulatory circuits of T cell function in cancer. Nat. Rev. Immunol..

[4] Zou W. (2006). Regulatory T cells, tumour immunity and immunotherapy. Nat. Rev. Immunol..

[5] Kennedy R., Celis E. (2008). Multiple roles for CD4+ T cells in anti-tumor immune responses. Immunol. Rev..

[6] Melssen M., Slingluff C.L. (2017). Vaccines targeting helper T cells for cancer immunotherapy. Curr. Opin. Immunol..

[7] Borst J., Ahrends T., Bąbała N., Melief C.J.M., Kastenmüller W. (2018). CD4+ T cell help in cancer immunology and immunotherapy. Nat. Rev. Immunol..

[8] Raskov H., Orhan A., Christensen J.P., Gögenur I. (2021). Cytotoxic CD8+ T cells in cancer and cancer immunotherapy. Br. J. Cancer.

[9] Raverdeau M., Cunningham S.P., Harmon C., Lynch L. (2019). γδ T cells in cancer: A small population of lymphocytes with big implications. Clin. Transl. Immunol..

[10] Bennstein S.B. (2018). Unraveling natural killer T-cells development. Front. Immunol..

[11] Toubal A., Nel I., Lotersztajn S., Lehuen A. (2019). Mucosal-associated invariant T cells and disease. Nat. Rev. Immunol..

[12] Bedoui S., Gebhardt T., Gasteiger G., Kastenmüller W. (2016). Parallels and differences between innate and adaptive lymphocytes. Nat. Immunol..

[13] Kronenberg M. (2005). Toward an understanding of NKT cell biology: Progress and paradoxes. Annu. Rev. Immunol..

[14] Van Kaer L., Postoak J.L., Wang C., Yang G., Wu L. (2019). Innate, innate-like and adaptive lymphocytes in the pathogenesis of MS and EAE. Cell. Mol. Immunol..

[15] Vantourout P., Hayday A. (2013). Six-of-the-best: Unique contributions of γδ T cells to immunology. Nat. Rev. Immunol..

[16] Chien Y.H., Meyer C., Bonneville M. (2014). γδ T cells: First line of defense and beyond. Annu. Rev. Immunol..

[17] Lantz O., Legoux F. (2018). MAIT cells: An historical and evolutionary perspective. Immunol. Cell Biol..

[18] Keller A.N., Corbett A.J., Wubben J.M., McCluskey J., Rossjohn J. (2017). MAIT cells and MR1-antigen recognition. Curr. Opin. Immunol..

[19] Mougiakakos D., Johansson C.C.C., Trocme E., All-Ericsson C., Economou M.A.A., Larsson O., Seregard S., Kiessling R. (2010). Intratumoral forkhead box p3-positive regulatory T cells predict poor survival in cyclooxygenase-2-positive uveal melanoma. Cancer.

[20] Piccirillo C.A.A. (2008). Regulatory T cells in health and disease. Cytokine.

[21] Maj T., Wang W., Crespo J., Zhang H., Wang W., Wei S., Zhao L., Vatan L., Shao I., Szeliga W. (2017). Oxidative stress controls regulatory T cell apoptosis and suppressor activity and PD-L1-blockade resistance in tumor. Nat. Immunol..

[22] Vanamee É.S.S., Faustman D.L.L. (2017). TNFR2: A Novel Target for Cancer Immunotherapy. Trends Mol. Med..

[23] Zeng G., Jin L., Ying Q., Chen H., Thembinkosi M.C.C., Yang C., Zhao J., Ji H., Lin S., Peng R. (2020). Regulatory T cells in cancer immunotherapy: Basic research outcomes and clinical directions. Cancer Manag. Res..

[24] Togashi Y., Shitara K., Nishikawa H. (2019). Regulatory T cells in cancer immunosuppression—Implications for anticancer therapy. Nat. Rev. Clin. Oncol..

[25] Atkins M.B.B., Clark J.I.I., Quinn D.I.I. (2017). Immune checkpoint inhibitors in advanced renal cell carcinoma: Experience to date and future directions. Ann. Oncol. Off. J. Eur. Soc. Med. Oncol..

[26] Foell J., Hewes B. (2007). T Cell Costimulatory and Inhibitory Receptors as Therapeutic Targets for Inducing Anti-Tumor Immunity. Curr. Cancer Drug Targets.

[27] Wei S.C., Duffy C.R., Allison J.P. (2018). Fundamental mechanisms of immune checkpoint blockade therapy. Cancer Discov..

[28] Pentcheva-Hoang T., Egen J.G., Wojnoonski K., Allison J.P. (2004). B7-1 and B7-2 selectively recruit CTLA-4 and CD28 to the immunological synapse. Immunity.

[29] Walunas T.L., Lenschow D.J., Bakker C.Y., Linsley P.S., Freeman G.J., Green J.M., Thompson C.B., Bluestone J.A. (1994). CTLA-4 can function as a negative regulator of T cell activation. Immunity.

[30] Brunner M.C., Chambers C.A., Chan F.K., Hanke J., Winoto A., Allison J.P. CTLA-4-Mediated inhibition of early events of T cell proliferation—PubMed. https://pubmed.ncbi.nlm.nih.gov/10229815/.

[31] Linsley P.S., Brady W., Urnes M., Grosmaire L.S., Damle N.K., Ledbetter J.A. (1991). CTLA4 is a second receptor for the b cell activation antigen B7. J. Exp. Med..

[32] Linsley P.S., Greene J.A.L., Brady W., Bajorath J., Ledbetter J.A., Peach R. (1994). Human B7-1 (CD80) and B7-2 (CD86) bind with similar avidities but distinct kinetics to CD28 and CTLA-4 receptors. Immunity.

[33] Lanier L.L., O’Fallon S., Somoza C., Phillips J.H., Linsley P.S., Okumura K., Ito D., Azuma M. (1995). CD80 (B7) and CD86 (B70) provide similar costimulatory signals for T cell proliferation, cytokine production, and generation of CTL. J. Immunol..

[34] Van Der Merwe P.A., Bodian D.L., Daenke S., Linsley P., Davis S.J. (1997). CD80 (B7-1) binds both CD28 and CTLA-4 with a low affinity and very fast kinetics. J. Exp. Med..

[35] Yi J., Kawabe T., Sprent J. (2020). New insights on T-cell self-tolerance. Curr. Opin. Immunol..

[36] Qureshi O.S., Zheng Y., Nakamura K., Attridge K., Manzotti C., Schmidt E.M., Baker J., Jeffery L.E., Kaur S., Briggs Z. (2011). Trans-endocytosis of CD80 and CD86: A molecular basis for the cell-extrinsic function of CTLA-4. Science.

[37] Bachmann M.F., Köhler G., Ecabert B., Mak T.W., Kopf M. Cutting Edge: Lymphoproliferative Disease in the Absence of CTLA-4 is Not T Cell Autonomous—PubMed. https://pubmed.ncbi.nlm.nih.gov/10415006/.

[38] Wing K., Onishi Y., Prieto-Martin P., Yamaguchi T., Miyara M., Fehervari Z., Nomura T., Sakaguchi S. (2008). CTLA-4 control over Foxp3+ regulatory T cell function. Science.

[39] Yang Y., Li X., Ma Z., Wang C., Yang Q., Byrne-Steele M., Hong R., Min Q., Zhou G., Cheng Y. (2021). CTLA-4 expression by B-1a B cells is essential for immune tolerance. Nat. Commun..

[40] Egen J.G., Allison J.P. (2002). Cytotoxic T lymphocyte antigen-4 accumulation in the immunological synapse is regulated by TCR signal strength. Immunity.

[41] Pagès F., Ragueneau M., Rottapel R., Truneh A., Nunes J., Imbert J., Olive D. (1994). Binding of phosphatidyl-inositol-3-OH kinase to CD28 is required for T-cell signalling. Nature.

[42] Kane L.P., Andres P.G., Howland K.C., Abbas A.K., Weiss A. (2001). Akt provides the CD28 costimulatory signal for up-regulation of IL-2 and IFN-γ but not TH2 cytokines. Nat. Immunol..

[43] Chambers C.A., Cado D., Truong T., Allison J.P. (1997). Thymocyte development is normal in CTLA-4-deficient mice. Proc. Natl. Acad. Sci. USA.

[44] Waterhouse P., Penninger J.M., Timms E., Wakeham A., Shahinian A., Lee K.P., Thompson C.B., Griesser H., Mak T.W. (1995). Lymphoproliferative disorders with early lethality in mice deficient in Ctla-4. Science.

[45] Tivol E.A., Borriello F., Schweitzer A.N., Lynch W.P., Bluestone J.A., Sharpe A.H. (1995). Loss of CTLA-4 leads to massive lymphoproliferation and fatal multiorgan tissue destruction, revealing a critical negative regulatory role of CTLA-4. Immunity.

[46] Friedline R.H., Brown D.S., Nguyen H., Kornfeld H., Lee J., Zhang Y., Appleby M., Der S.D., Kang J., Chambers C.A. (2009). CD4+ regulatory T cells require CTLA-4 for the maintenance of systemic tolerance. J. Exp. Med..

[47] Quezada S.A., Peggs K.S., Curran M.A., Allison J.P. (2006). CTLA4 blockade and GM-CSF combination immunotherapy alters the intratumor balance of effector and regulatory T cells. J. Clin. Investig..

[48] Sharma A., Subudhi S.K., Blando J., Scutti J., Vence L., Wargo J., Allison J.P., Ribas A., Sharma P. (2019). Anti-CTLA-4 immunotherapy does not deplete Foxp3 þ regulatory T cells (Tregs) in human cancers. Clin. Cancer Res..

[49] Jain N., Nguyen H., Chambers C., Kang J. (2010). Dual function of CTLA-4 in regulatory T cells and conventional T cells to prevent multiorgan autoimmunity. Proc. Natl. Acad. Sci. USA.

[50] Corse E., Allison J.P. (2012). Cutting Edge: CTLA-4 on Effector T Cells Inhibits In Trans. J. Immunol..

[51] Paterson A.M., Lovitch S.B., Sage P.T., Juneja V.R., Lee Y., Trombley J.D., Arancibia-Cárcamo C.V., Sobel R.A., Rudensky A.Y., Kuchroo V.K. (2015). Deletion of CTLA-4 on regulatory T cells during adulthood leads to resistance to autoimmunity. J. Exp. Med..

[52] Doyle A.M., Mullen A.C., Villarino A.V., Hutchins A.S., High F.A., Lee H.W., Thompson C.B., Reiner S.L. (2001). Induction of cytotoxic T lymphocyte antigen 4 (CTLA-4) restricts clonal expansion of helper T cells. J. Exp. Med..

[53] Egen J.G., Kuhns M.S., Allison J.P. (2002). CTLA-4: New insights into its biological function and use in tumor immunotherapy. Nat. Immunol..

[54] Busch D.H., Pamer E.G. (1999). T cell affinity maturation by selective expansion during infection. J. Exp. Med..

[55] Savage P.A., Boniface J.J., Davis M.M. (1999). A kinetic basis for T cell receptor repertoire selection during an immune response. Immunity.

[56] Metzler W.J., Bajorath J., Fenderson W., Shaw S.Y., Constantine K.L., Naemura J., Leytze G., Peach R.J., Lavoie T.B., Mueller L. (1997). Solution structure of human CTLA-4 and delineation of a CD80/CD86 binding site conserved in CD28. Nat. Struct. Biol..

[57] Yu C., Sonnen A.F.P., George R., Dessailly B.H., Stagg L.J., Evans E.J., Orengo C.A., Stuart D.I., Ladbury J.E., Ikemizu S. (2011). Rigid-body ligand recognition drives cytotoxic T-lymphocyte antigen 4 (CTLA-4) receptor triggering. J. Biol. Chem..

[58] Stamper C.C., Zhang Y., Tobin J.F., Erbe D.V., Ikemizu S., Davis S.J., Stahl M.L., Seehra J., Somers W.S., Mosyak L. (2001). Crystal structure of the B7-1/CTLA-4 complex that inhibits human immune responses. Nature.

[59] Schwartz J.C.D., Zhang X., Fedorov A.A., Nathenson S.G., Almo S.C. (2001). Structural basis for co-stimulation by the human CTLA-4/B7-2 complex. Nature.

[60] Zhang F., Qi X., Wang X., Wei D., Wu J., Feng L., Cai H., Wang Y., Zeng N., Xu T. (2017). Structural basis of the therapeutic anti-PD-L1 antibody atezolizumab. Oncotarget.

[61] Ramagopal U.A., Liu W., Garrett-Thomson S.C., Bonanno J.B., Yan Q., Srinivasan M., Wong S.C., Bell A., Mankikar S., Rangan V.S. (2017). Structural basis for cancer immunotherapy by the first-in-class checkpoint inhibitor ipilimumab. Proc. Natl. Acad. Sci. USA.

[62] He M., Chai Y., Qi J., Zhang C.W.H., Tong Z., Shi Y., Yan J., Tan S., Gao G.F. (2017). Remarkably similar CTLA-4 binding properties of therapeutic ipilimumab and tremelimumab antibodies. Oncotarget.

[63] Hueber A.J., Matzkies F.G., Rahmeh M., Manger B., Kalden J.R., Nagel T. (2006). CTLA-4 lacking the cytoplasmic domain costimulates IL-2 production in T-cell hybridomas. Immunol. Cell Biol..

[64] Rudd C.E. (2008). The reverse stop-signal model for CTLA4 function. Nat. Rev. Immunol..

[65] Thompson C.B., Allison J.P. (1997). The emerging role of CTLA-4 as an immune attenuator. Immunity.

[66] Waterhouse P., Marengère L.E.M., Mittrücker H.W., Mak T.W. (1996). CTLA-4, a negative regulator of T-Lymphocyte activation. Immunol. Rev..

[67] Marengère L.E.M., Waterhouse P., Duncan G.S., Mittrücker H.W., Feng G.S., Mak T.W. (1996). Regulation of T cell receptor signaling by tyrosine phosphatase SYP association with CTLA-4. Science.

[68] Wang H., Sun Y., Zhou X., Chen C., Jiao L., Li W., Gou S., Li Y., Du J., Chen G. (2020). CD47/SIRPα blocking peptide identification and synergistic effect with irradiation for cancer immunotherapy. J. Immunother. Cancer.

[69] Kaspar A.A., Reichert J.M. (2013). Future directions for peptide therapeutics development. Drug Discov. Today.

[70] Xie J., Si X., Gu S., Wang M., Shen J., Li H., Li D., Fang Y., Liu C., Zhu J. (2017). Allosteric Inhibitors of SHP2 with Therapeutic Potential for Cancer Treatment. J. Med. Chem..

[71] Song K., Liu X., Huang W., Lu S., Shen Q., Zhang L., Zhang J. (2017). Improved Method for the Identification and Validation of Allosteric Sites. J. Chem. Inf. Model..

[72] Roca C., Requena C., Sebastián-Pérez V., Malhotra S., Radoux C., Pérez C., Martinez A., Antonio Páez J., Blundell T.L., Campillo N.E. (2018). Identification of new allosteric sites and modulators of AChE through computational and experimental tools. J. Enzyme Inhib. Med. Chem..

[73] Sakaguchi S., Sakaguchi N., Asano M., Itoh M., Toda M. (1995). Immunologic self-tolerance maintained by activated T cells expressing IL-2 receptor alpha-chains (CD25). Breakdown of a single mechanism of self-tolerance causes various autoimmune diseases. J. Immunol..

[74] Bennett C.L., Christie J., Ramsdell F., Brunkow M.E., Ferguson P.J., Whitesell L., Kelly T.E., Saulsbury F.T., Chance P.F., Ochs H.D. (2001). The immune dysregulation, polyendocrinopathy, enteropathy, X-linked syndrome (IPEX) is caused by mutations of FOXP3. Nat. Genet..

[75] Fontenot J.D., Gavin M.A., Rudensky A.Y. (2017). Foxp3 programs the development and function of CD4+CD25+ regulatory T cells. J. Immunol..

[76] Brunkow M.E., Jeffery E.W., Hjerrild K.A., Paeper B., Clark L.B., Yasayko S.A., Wilkinson J.E., Galas D., Ziegler S.F., Ramsdell F. (2001). Disruption of a new forkhead/winged-helix protein, scurfin, results in the fatal lymphoproliferative disorder of the scurfy mouse. Nat. Genet..

[77] Linterman M.A., Pierson W., Lee S.K., Kallies A., Kawamoto S., Rayner T.F., Srivastava M., Divekar D.P., Beaton L., Hogan J.J. (2011). Foxp3+ follicular regulatory T cells control the germinal center response. Nat. Med..

[78] Koch M.A., Tucker-Heard G., Perdue N.R., Killebrew J.R., Urdahl K.B., Campbell D.J. (2009). The transcription factor T-bet controls regulatory T cell homeostasis and function during type 1 inflammation. Nat. Immunol..

[79] Chung Y., Tanaka S., Chu F., Nurieva R.I., Martinez G.J., Rawal S., Wang Y.H., Lim H., Reynolds J.M., Zhou X.H. (2011). Follicular regulatory T cells expressing Foxp3 and Bcl-6 suppress germinal center reactions. Nat. Med..

[80] Chaudhry A., Rudra D., Treuting P., Samstein R.M., Liang Y., Kas A., Rudensky A.Y. (2009). CD4+ regulatory T cells control TH17 responses in a stat3-dependent manner. Science.

[81] Wing K., Sakaguchi S. (2010). Regulatory T cells exert checks and balances on self tolerance and autoimmunity. Nat. Immunol..

[82] Sakaguchi S., Miyara M., Costantino C.M., Hafler D.A. (2010). FOXP3 + regulatory T cells in the human immune system. Nat. Rev. Immunol..

[83] Togashi Y., Nishikawa H. (2017). Regulatory T cells: Molecular and cellular basis for immunoregulation. Current Topics in Microbiology and Immunology.

[84] Hori S., Nomura T., Sakaguchi S. (2017). Control of regulatory T cell development by the transcription factor Foxp3. J. Immunol..

[85] Khattri R., Cox T., Yasayko S.A., Ramsdell F. (2017). An essential role for Scurfin in CD4+CD25+T regulatory cells. J. Immunol..

[86] Steinbrink K., Wölfl M., Jonuleit H., Knop J., Enk A.H.H. (1997). Induction of tolerance by IL-10-treated dendritic cells. J. Immunol..

[87] Collison L.W.W., Workman C.J.J., Kuo T.T.T., Boyd K., Wang Y., Vignali K.M.M., Cross R., Sehy D., Blumberg R.S.S., Vignali D.A.A.A.A. (2007). The inhibitory cytokine IL-35 contributes to regulatory T-cell function. Nature.

[88] Turnis M.E.E., Sawant D.V.V., Szymczak-Workman A.L.L., Andrews L.P.P., Delgoffe G.M.M., Yano H., Beres A.J.J., Vogel P., Workman C.J.J., Vignali D.A. (2016). Interleukin-35 Limits Anti-Tumor Immunity. Immunity.

[89] Jarnicki A.G.G., Lysaght J., Todryk S., Mills K.H.G.H.G. (2006). Suppression of Antitumor Immunity by IL-10 and TGF-β-Producing T Cells Infiltrating the Growing Tumor: Influence of Tumor Environment on the Induction of CD4+ and CD8+ Regulatory T Cells. J. Immunol..

[90] Takahashi T., Kuniyasu Y., Toda M., Sakaguchi N., Itoh M., Iwata M., Shimizu J., Sakaguchi S. (1998). Immunologic self-tolerance maintained by CD25+CD4+ naturally anergic and suppressive T cells: Induction of autoimmune disease by breaking their anergic/suppressive state. Int. Immunol..

[91] Thornton A.M.M., Shevach E.M.M. (1998). CD4+CD25+ immunoregulatory T cells suppress polyclonal T cell activation in vitro by inhibiting interleukin 2 production. J. Exp. Med..

[92] Perez V.L.L., Van Parijs L., Biuckians A., Zheng X.X.X., Strom T.B.B., Abbas A.K.K. (1997). Induction of peripheral T cell tolerance in vivo requires CTLA-4 engagement. Immunity.

[93] Onizuka S., Tawara I., Shimizu J., Sakaguchi S., Fujita T., Nakayama E. (1999). Tumor rejection by in vivo administration of anti-CD25 (interleukin-2 receptor α) monoclonal antibody. Cancer Res..

[94] Shimizu J., Yamazaki S., Sakaguchi S. (1999). Induction of tumor immunity by removing CD25+CD4+ T cells: A common basis between tumor immunity and autoimmunity—PubMed. J. Immunol..

[95] Lodish H., Berk A., Zipursky S.L., Matsudaira P., Baltimore D., Darnell J. (2000). Proto-Oncogenes and Tumor-Suppressor Genes. Molecular Cell Biology.

[96] Saito T., Nishikawa H., Wada H., Nagano Y., Sugiyama D., Atarashi K., Maeda Y., Hamaguchi M., Ohkura N., Sato E. (2016). Two FOXP3 + CD4 + T cell subpopulations distinctly control the prognosis of colorectal cancers. Nat. Med..

[97] Tada Y., Togashi Y., Kotani D., Kuwata T., Sato E., Kawazoe A., Doi T., Wada H., Nishikawa H., Shitara K. (2018). Targeting VEGFR2 with Ramucirumab strongly impacts effector/activated regulatory T cells and CD8+ T cells in the tumor microenvironment. J. Immunother. Cancer.

[98] Fridman W.H., Pagès F., Sautès-Fridman C., Galon J. (2012). The immune contexture in human tumours: Impact on clinical outcome. Nat. Rev. Cancer.

[99] Shang B., Liu Y., Jiang S.J., Liu Y. (2015). Prognostic value of tumor-infiltrating FoxP3+ regulatory T cells in cancers: A systematic review and meta-analysis. Sci. Rep..

[100] Kryczek I., Wu K., Zhao E., Wei S., Vatan L., Szeliga W., Huang E., Greenson J., Chang A., Roliński J. (2011). IL-17 + Regulatory T Cells in the Microenvironments of Chronic Inflammation and Cancer. J. Immunol..

[101] Kryczek I., Liu R., Wang G., Wu K., Shu X., Szeliga W., Vatan L., Finlayson E., Huang E., Simeone D. (2009). FOXP3 defines regulatory T cells in human tumor and autoimmune disease. Cancer Res..

[102] Colbeck E.J., Jones E., Hindley J.P., Smart K., Schulz R., Browne M., Cutting S., Williams A., Parry L., Godkin A. (2017). Treg depletion licenses T cell–driven HEV neogenesis and promotes tumor destruction. Cancer Immunol. Res..

[103] Hindley J.P., Jones E., Smart K., Bridgeman H., Lauder S.N., Ondondo B., Cutting S., Ladell K., Wynn K.K., Withers D. (2012). T-cell trafficking facilitated by high endothelial venules is required for tumor control after regulatory T-cell depletion. Cancer Res..

[104] Sugiyama D., Nishikawa H., Maeda Y., Nishioka M., Tanemura A., Katayama I., Ezoe S., Kanakura Y., Sato E., Fukumori Y. (2013). Anti-CCR4 mAb selectively depletes effector-Type FoxP3+CD4+ regulatory T cells, evoking antitumor immune responses in humans. Proc. Natl. Acad. Sci. USA.

[105] Facciabene A., Peng X., Hagemann I.S., Balint K., Barchetti A., Wang L.P., Gimotty P.A., Gilks C.B., Lal P., Zhang L. (2011). Tumour hypoxia promotes tolerance and angiogenesis via CCL28 and T reg cells. Nature.

[106] Togashi Y., Nishikawa H. (2017). Suppression from beyond the grave. Nat. Immunol..

[107] Nishikawa H., Kato T., Tawara I., Saito K., Ikeda H., Kuribayashi K., Allen P.M., Schreiber R.D., Sakaguchi S., Old L.J. (2005). Definition of target antigens for naturally occurring CD4+ CD25+ regulatory T cells. J. Exp. Med..

[108] Ghiringhelli F., Puig P.E., Roux S., Parcellier A., Schmitt E., Solary E., Kroemer G., Martin F., Chauffert B., Zitvogel L. (2005). Tumor cells convert immature myeloid dendritic cells into TGF-β-secreting cells inducing CD4 +CD25 + regulatory T cell proliferation. J. Exp. Med..

[109] Hindley J.P., Ferreira C., Jones E., Lauder S.N., Ladell K., Wynn K.K., Betts G.J., Singh Y., Price D.A., Godkin A.J. (2011). Analysis of the T-cell receptor repertoires of tumor-infiltrating conventional and regulatory T cells reveals no evidence for conversion in carcinogen-induced tumors. Cancer Res..

[110] Sainz-Perez A., Lim A., Lemercier B., Leclerc C. (2012). The T-cell receptor repertoire of tumor-infiltrating regulatory T lymphocytes is skewed toward public sequences. Cancer Res..

[111] Morikawa H., Ohkura N., Vandenbon A., Itoh M., Nagao-Sato S., Kawaji H., Lassmann T., Carninci P., Hayashizaki Y., Forrest A.R.R. (2014). Differential roles of epigenetic changes and Foxp3 expression in regulatory T cell-specific transcriptional regulation. Proc. Natl. Acad. Sci. USA.

[112] Wei G., Wei L., Zhu J., Zang C., Hu-Li J., Yao Z., Cui K., Kanno Y., Roh T.Y., Watford W.T. (2009). Global Mapping of H3K4me3 and H3K27me3 Reveals Specificity and Plasticity in Lineage Fate Determination of Differentiating CD4+ T Cells. Immunity.

[113] Ohkura N., Hamaguchi M., Morikawa H., Sugimura K., Tanaka A., Ito Y., Osaki M., Tanaka Y., Yamashita R., Nakano N. (2012). T Cell Receptor Stimulation-Induced Epigenetic Changes and Foxp3 Expression Are Independent and Complementary Events Required for Treg Cell Development. Immunity.

[114] Curiel T.J., Coukos G., Zou L., Alvarez X., Cheng P., Mottram P., Evdemon-Hogan M., Conejo-Garcia J.R., Zhang L., Burow M. (2004). Specific recruitment of regulatory T cells in ovarian carcinoma fosters immune privilege and predicts reduced survival. Nat. Med..

[115] Wei S., Kryczek I., Edwards R.P., Zou L., Szeliga W., Banerjee M., Cost M., Cheng P., Chang A., Redman B. (2007). Interleukin-2 administration alters the CD4+FOXP3+ T-cell pool and tumor trafficking in patients with ovarian carcinoma. Cancer Res..

[116] Tan M.C.B., Goedegebuure P.S., Belt B.A., Flaherty B., Sankpal N., Gillanders W.E., Eberlein T.J., Hsieh C.-S., Linehan D.C. (2009). Disruption of CCR5-Dependent Homing of Regulatory T Cells Inhibits Tumor Growth in a Murine Model of Pancreatic Cancer. J. Immunol..

[117] Hoelzinger D.B., Smith S.E., Mirza N., Dominguez A.L., Manrique S.Z., Lustgarten J. (2010). Blockade of CCL1 Inhibits T Regulatory Cell Suppressive Function Enhancing Tumor Immunity without Affecting T Effector Responses. J. Immunol..

[118] Sen D.R., Kaminski J., Barnitz R.A., Kurachi M., Gerdemann U., Yates K.B., Tsao H.W., Godec J., LaFleur M.W., Brown F.D. (2016). The epigenetic landscape of T cell exhaustion. Science.

[119] Spranger S., Spaapen R.M., Zha Y., Williams J., Meng Y., Ha T.T., Gajewski T.F. (2013). Up-regulation of PD-L1, IDO, and Tregs in the melanoma tumor microenvironment is driven by CD8+ T cells. Sci. Transl. Med..

[120] Williams J.B., Horton B.L., Zheng Y., Duan Y., Powell J.D., Gajewski T.F. (2017). The EGR2 targets LAG-3 and 4-1BB describe and regulate dysfunctional antigen-specific CD8+ T cells in the tumor microenvironment. J. Exp. Med..

[121] Zakiryanova G.K., Wheeler S., Shurin M.R. (2018). Oncogenes in immune cells as potential therapeutic targets. ImmunoTargets Ther..

[122] Spranger S., Bao R., Gajewski T.F. (2015). Melanoma-intrinsic β-catenin signalling prevents anti-tumour immunity. Nature.

[123] Peng W., Chen J.Q., Liu C., Malu S., Creasy C., Tetzlaff M.T., Xu C., McKenzie J.A., Zhang C., Liang X. (2016). Loss of PTEN promotes resistance to T cell–mediated immunotherapy. Cancer Discov..

[124] Spranger S., Gajewski T.F. (2018). Impact of oncogenic pathways on evasion of antitumour immune responses. Nat. Rev. Cancer.

[125] Hamarsheh S., Groß O., Brummer T., Zeiser R. (2020). Immune modulatory effects of oncogenic KRAS in cancer. Nat. Commun..

[126] Jiang H., Hegde S., Knolhoff B.L., Zhu Y., Herndon J.M., Meyer M.A., Nywening T.M., Hawkins W.G., Shapiro I.M., Weaver D.T. (2016). Targeting focal adhesion kinase renders pancreatic cancers responsive to checkpoint immunotherapy. Nat. Med..

[127] Serrels A., Lund T., Serrels B., Byron A., McPherson R.C., Von Kriegsheim A., Gómez-Cuadrado L., Canel M., Muir M., Ring J.E. (2015). Nuclear FAK Controls Chemokine Transcription, Tregs, and Evasion of Anti-tumor Immunity. Cell.

[128] Pace L., Tempez A., Arnold-Schrauf C., Lemaitre F., Bousso P., Fetler L., Sparwasser T., Amigorena S. (2012). Regulatory T cells increase the avidity of primary CD8+ T cell responses and promote memory. Science.

[129] Maeda Y., Nishikawa H., Sugiyama D., Ha D., Hamaguchi M., Saito T., Nishioka M., Wing J.B., Adeegbe D., Katayama I. (2014). Detection of self-reactive CD8+ T cells with an anergic phenotype in healthy individuals. Science.

[130] Snyder A., Makarov V., Merghoub T., Yuan J., Zaretsky J.M., Desrichard A., Walsh L.A., Postow M.A., Wong P., Ho T.S. (2014). Genetic Basis for Clinical Response to CTLA-4 Blockade in Melanoma. N. Engl. J. Med..

[131] Rizvi N.A., Hellmann M.D., Snyder A., Kvistborg P., Makarov V., Havel J.J., Lee W., Yuan J., Wong P., Ho T.S. (2015). Mutational landscape determines sensitivity to PD-1 blockade in non-small cell lung cancer. Science.

[132] Hodi F.S., O’Day S.J., McDermott D.F., Weber R.W., Sosman J.A., Haanen J.B., Gonzalez R., Robert C., Schadendorf D., Hassel J.C. (2010). Improved Survival with Ipilimumab in Patients with Metastatic Melanoma. N. Engl. J. Med..

[133] Robert C., Thomas L., Bondarenko I., O’Day S., Weber J., Garbe C., Lebbe C., Baurain J.-F., Testori A., Grob J.-J. (2011). Ipilimumab plus Dacarbazine for Previously Untreated Metastatic Melanoma. N. Engl. J. Med..

[134] Fellne C. (2012). Ipilimumab (Yervoy) prolongs survival in advanced melanoma: Serious side effects and a hefty price tag may limit its use. Pharm. Ther..

[135] Zhang Y., Du X., Liu M., Tang F., Zhang P., Ai C., Fields J.K., Sundberg E.J., Latinovic O.S., Devenport M. (2019). Hijacking antibody-induced CTLA-4 lysosomal degradation for safer and more effective cancer immunotherapy. Cell Res..

[136] Ji D., Song C., Li Y., Xia J., Wu Y., Jia J., Cui X., Yu S., Gu J. (2020). Combination of radiotherapy and suppression of Tregs enhances abscopal antitumor effect and inhibits metastasis in rectal cancer. J. Immunother. Cancer.

[137] Qu Q., Zhai Z., Xu J., Li S., Chen C., Lu B. (2020). IL36 Cooperates with Anti-CTLA-4 mAbs to Facilitate Antitumor Immune Responses. Front. Immunol..

[138] Mihic-Probst D., Reinehr M., Dettwiler S., Kolm I., Britschgi C., Kudura K., Maggio E.M., Lenggenhager D., Rushing E.J. (2020). The role of macrophages type 2 and T-regs in immune checkpoint inhibitor related adverse events. Immunobiology.

[139] Sun N.Y., Chen Y.L., Lin H.W., Chiang Y.C., Chang C.F., Tai Y.J., Chen C.A., Sun W.Z., Chien C.L., Cheng W.F. (2019). Immune checkpoint Ab enhances the antigen-specific anti-tumor effects by modulating both dendritic cells and regulatory T lymphocytes. Cancer Lett..

[140] Kvarnhammar A.M., Veitonmäki N., Hägerbrand K., Dahlman A., Smith K.E., Fritzell S., Von Schantz L., Thagesson M., Werchau D., Smedenfors K. (2019). The CTLA-4 x OX40 bispecific antibody ATOR-1015 induces anti-tumor effects through tumor-directed immune activation. J. Immunother. Cancer.

[141] Pai C.C.S., Simons D.M., Lu X., Evans M., Wei J., Wang Y.H., Chen M., Huang J., Park C., Chang A. (2019). Tumor-conditional anti-CTLA4 uncouples antitumor efficacy from immunotherapy-related toxicity. J. Clin. Investig..

[142] Morris Z.S., Guy E.I., Werner L.R., Carlson P.M., Heinze C.M., Kler J.S., Busche S.M., Jaquish A.A., Sriramaneni R.N., Carmichael L.L. (2018). Tumor-specific inhibition of in situ vaccination by distant untreated tumor sites. Cancer Immunol. Res..

[143] Duperret E.K., Wise M.C., Trautz A., Villarreal D.O., Ferraro B., Walters J., Yan J., Khan A., Masteller E., Humeau L. (2018). Synergy of Immune Checkpoint Blockade with a Novel Synthetic Consensus DNA Vaccine Targeting TERT. Mol. Ther..

[144] Tang F., Du X., Liu M., Zheng P., Liu Y. (2018). Anti-CTLA-4 antibodies in cancer immunotherapy: Selective depletion of intratumoral regulatory T cells or checkpoint blockade?. Cell Biosci..

[145] Son C.H., Bae J., Lee H.R., Yang K., Park Y.S. (2017). Enhancement of antitumor immunity by combination of anti-CTLA-4 antibody and radioimmunotherapy through the suppression of Tregs. Oncol. Lett..

[146] Schwarz C., Unger L., Mahr B., Aumayr K., Regele H., Farkas A.M., Hock K., Pilat N., Wekerle T. (2016). The Immunosuppressive Effect of CTLA4 Immunoglobulin Is Dependent on Regulatory T Cells at Low But Not High Doses. Am. J. Transplant..

[147] Marabelle A., Kohrt H., Sagiv-Barfi I., Ajami B., Axtell R.C., Zhou G., Rajapaksa R., Green M.R., Torchia J., Brody J. (2013). Depleting tumor-specific Tregs at a single site eradicates disseminated tumors. J. Clin. Invest..

[148] Du X., Tang F., Liu M., Su J., Zhang Y., Wu W., Devenport M., Lazarski C.A., Zhang P., Wang X. (2018). A reappraisal of CTLA-4 checkpoint blockade in cancer immunotherapy. Cell Res..

[149] Sandin L.C., Eriksson F., Ellmark P., Loskog A.S.I., Tötterman T.H., Mangsbo S.M. (2014). Local CTLA4 blockade effectively restrains experimental pancreatic adenocarcinoma growth in vivo. Oncoimmunology.

[150] Kavanagh B., O’Brien S., Lee D., Hou Y., Weinberg V., Rini B., Allison J.P., Small E.J., Fong L. (2008). CTLA4 blockade expands FoxP3+ regulatory and activated effector CD4 + T cells in a dose-dependent fashion. Blood.

[151] Francisco L.M., Salinas V.H., Brown K.E., Vanguri V.K., Freeman G.J., Kuchroo V.K., Sharpe A.H. (2009). PD-L1 regulates the development, maintenance, and function of induced regulatory T cells. J. Exp. Med..

[152] Li C., Jiang P., Wei S., Xu X., Wang J. (2020). Regulatory T cells in tumor microenvironment: New mechanisms, potential therapeutic strategies and future prospects. Mol. Cancer.

[153] Khan S., Burt D.J., Ralph C., Thistlethwaite F.C., Hawkins R.E., Elkord E. (2011). Tremelimumab (anti-CTLA4) mediates immune responses mainly by direct activation of T effector cells rather than by affecting T regulatory cells. Clin. Immunol..

[154] Kumar P., Saini S., Prabhakar B.S. (2020). Cancer immunotherapy with check point inhibitor can cause autoimmune adverse events due to loss of Treg homeostasis. Semin. Cancer Biol..

